# Supplementary figures and images for: The use of respiratory rate-oxygenation index to predict failure of high-flow nasal cannula in patients with coronavirus disease 2019-associated acute respiratory distress syndrome: A retrospective study
Source: PLoS One. 2023 Jun 21;18(6):e0287432. doi: 10.1371/journal.pone.0287432 (PMC10284391; doi:10.1371/journal.pone.0287432)

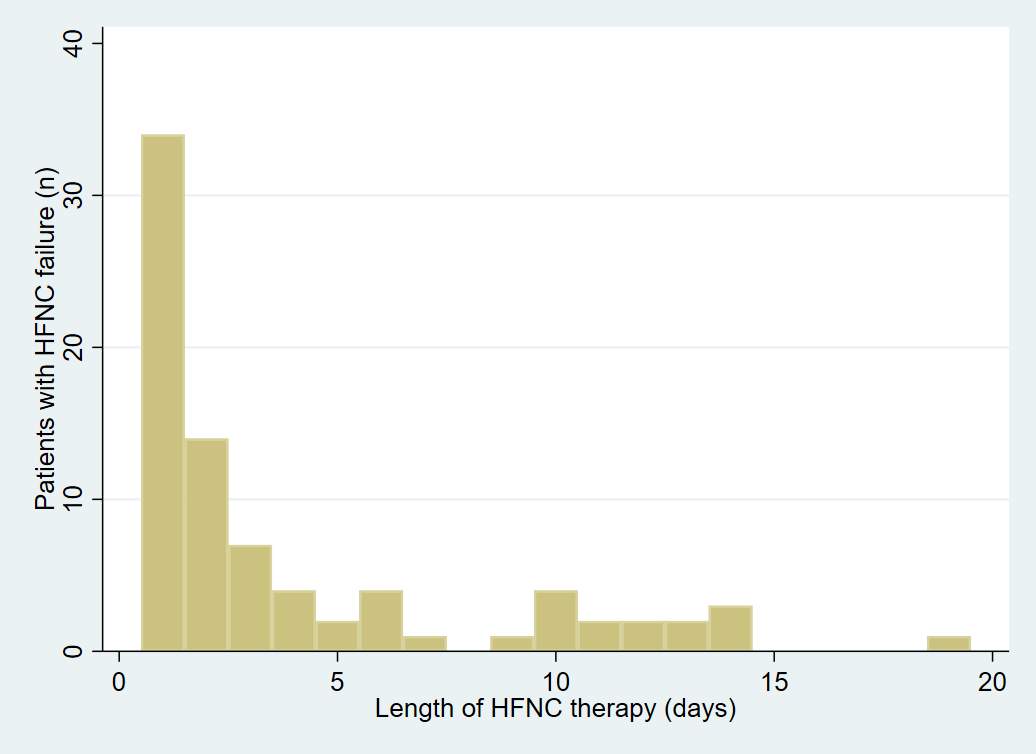

Supplement: S1 Fig — (TIFF) [file pone.0287432.s001.tiff]

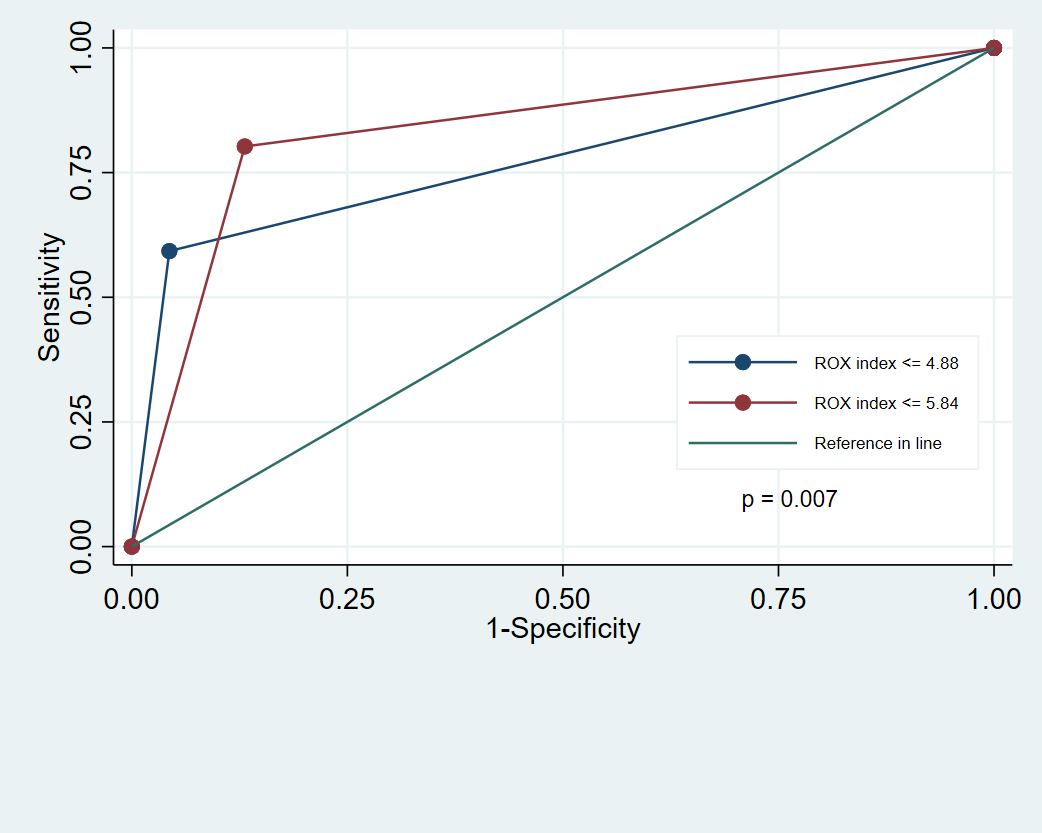

Supplement: S2 Fig — (TIFF) [file pone.0287432.s002.tiff]
