# Supplementary material for: The use of respiratory rate-oxygenation index to predict failure of high-flow nasal cannula in patients with coronavirus disease 2019-associated acute respiratory distress syndrome: A retrospective study
Source: PLoS One. 2023 Jun 21;18(6):e0287432. doi: 10.1371/journal.pone.0287432 (PMC10284391; doi:10.1371/journal.pone.0287432)
Supplement: S1 Table — (DOCX) [file pone.0287432.s003.docx]

**S1 Table Respiratory parameters within 7 days after the initiation of HFNC treatment**

| Respiratory parameters | Time | No. of intubation  (n=81) | HFNC failure | HFNC success | p value |
| --- | --- | --- | --- | --- | --- |
| ROX index | 1^st^ day | n=34 | 4.92 (3.92-5.75) | 7.92 (6.60-9.79) | <0.01 |
|  | 2^nd^ day | n=14 | 3.88 (2.74-4.31) | 8.25 (6.88-11.36) | <0.01 |
|  | 3^rd^ day | n=7 | 5.42 (4.74-5.83) | 8.55 (7.07-11.88) | <0.01 |
|  | 4^th^ day | n=4 | 4.38 (4.15-4.64) | 9.20 (7.27-12.00) | <0.01 |
|  | 5^th^ day | n=2 | 3.64 (3.53-3.75) | 9.20 (7.20-11.75) | 0.02 |
|  | 6^th^ day | n=4 | 5.76 (4.39-6.42) | 9.00 (7.05-11.75) | 0.01 |
|  | 7^th^ day | n=1 | 4.27 (4.27-4.27) | 8.78 (6.93-12.00) | 0.09 |
| SpO2/FiO2 | 1^st^ day | n=34 | 97 (97-158) | 163 (157-200) | <0.01 |
|  | 2^nd^ day | n=14 | 113 (100-120) | 167 (157-238) | <0.01 |
|  | 3^rd^ day | n=7 | 125 (104-160) | 167 (157-240) | <0.01 |
|  | 4^th^ day | n=4 | 102 (100-107) | 190 (157-243) | <0.01 |
|  | 5^th^ day | n=2 | 106 (99-113) | 186 (157-238) | 0.02 |
|  | 6^th^ day | n=4 | 133 (101-160) | 167 (155-238) | 0.03 |
|  | 7^th^ day | n=1 | 94 (94-94) | 165 (150-240) | 0.09 |
| FiO2 | 1^st^ day | n=34 | 0.80 (0.60-1.00) | 0.60 (0.50-0.60) | <0.01 |
|  | 2^nd^ day | n=14 | 0.80 (0.80-0.90) | 0.60 (0.40-0.60) | <0.01 |
|  | 3^rd^ day | n=7 | 0.80 (0.60-0.90) | 0.60 (0.40-0.60) | <0.01 |
|  | 4^th^ day | n=4 | 0.85 (0.80-0.95) | 0.50 (0.40-0.60) | <0.01 |
|  | 5^th^ day | n=2 | 0.85 (0.80-0.90) | 0.50 (0.40-0.60) | 0.02 |
|  | 6^th^ day | n=4 | 0.70 (0.60-0.90) | 0.50 (0.40-0.60) | 0.04 |
|  | 7^th^ day | n=1 | 1.00 (1.00-1.00) | 0.60 (0.40-0.60) | 0.08 |
| RR | 1^st^ day | n=34 | 25 (22-30) | 20 (20-24) | <0.01 |
|  | 2^nd^ day | n=14 | 30 (26-36) | 20 (20-22) | <0.01 |
|  | 3^rd^ day | n=7 | 26 (22-28) | 20 (20-22) | <0.01 |
|  | 4^th^ day | n=4 | 24 (23-24) | 20 (20-22) | 0.01 |
|  | 5^th^ day | n=2 | 29 (28-30) | 20 (20-22) | 0.02 |
|  | 6^th^ day | n=4 | 25 (22-27) | 20 (20-22) | 0.02 |
|  | 7^th^ day | n=1 | 22 (22-22) | 20 (19-22) | 0.33 |
| SpO_2_ | 1^st^ day | n=34 | 94 (91-96) | 96 (95-99) | <0.01 |
|  | 2^nd^ day | n=14 | 92 (90-96) | 96 (95-98) | <0.01 |
|  | 3^rd^ day | n=7 | 96 (90-99) | 95 (96-98) | 0.52 |
|  | 4^th^ day | n=4 | 91 (84-96) | 96 (94-98) | 0.08 |
|  | 5^th^ day | n=2 | 90 (89-90) | 96 (95-98) | 0.02 |
|  | 6^th^ day | n=4 | 92 (90-96) | 97 (95-99) | 0.07 |
|  | 7^th^ day | n=1 | 94 (94-94) | 96 (95-98) | 0.31 |
