# Supplementary material for: The use of respiratory rate-oxygenation index to predict failure of high-flow nasal cannula in patients with coronavirus disease 2019-associated acute respiratory distress syndrome: A retrospective study
Source: PLoS One. 2023 Jun 21;18(6):e0287432. doi: 10.1371/journal.pone.0287432 (PMC10284391; doi:10.1371/journal.pone.0287432)
Supplement: S1 File — (DOCX) [file pone.0287432.s004.docx]

**- Study Protocol -**

**Validation of ROX index score as a prediction for high-flow nasal cannula failure in COVID-19 patients.**

Sujaree Poopipatpab1, Pruchwilai Nuchpramool1, Piyarat Phairatwet2, Todsapol Lertwattanachai3, Konlawij Trongtrakul4*

1 Department of Anesthesiology, Faculty of Medicine Vajira Hospital, Navamindradhiraj University, Bangkok, Thailand

2 Department of Internal Medicine, Faculty of Medicine Vajira Hospital, Navamindradhiraj University, Bangkok, Thailand

3 Department of Pharmacology, Faculty of Medicine Vajira Hospital, Navamindradhiraj University, Bangkok, Thailand

4 Department of Internal Medicine, Faculty of Medicine, Chiang Mai University, Chiang Mai, Thailand

**Introduction**

The novel coronavirus disease (COVID-19) outbreak has been rapidly spreading around the world, caused by severe acute respiratory syndrome coronavirus 2 (SAR-CoV2) infection. Previous studies demonstrated that 3-7% of these patients were associated with acute respiratory failure which requires respiratory support [1]. The Surviving Sepsis Campaign: Guidelines Recommend that patients with COVID-19 should be closely monitored in those patients who commenced on a non-invasive positive pressure ventilator (NIPPV) or high-flow nasal cannula (HFNC) [2]. When the patient’s respiratory status worsens, this patient usually requires an invasive mechanical ventilator [2]. Deciding to intubate in COVID-19 patients with acute respiratory failure should be based on the levels of oxygen saturation or may be followed by other clinical signs including tachypnea, use of respiratory accessory muscles, tracheal tugging, nasal flaring, or gasping. If patients with impending respiratory failure are intubated at the proper time, they may experience less self-inflicted lung injury [3]. If those patients exposure to delayed intubation, there might be encountered an increased length of intensive care unit stay, prolonged mechanical ventilation, increased morbidity, and increased mortality [4].

The ROX index is a non-invasive tool that can be used at the bedside, and can be calculated from the ratio of oxygen saturation (SpO2)/fractional inspired oxygen (FiO2)-to-respiratory rate (RR). Roca O. et al study presented the accuracy of the ROX index at a value of greater than 4.88 at 12 hours after HFNC treatment was associated with a lower risk of endotracheal tube (ET) intubation in non-COVID-19 associated acute respiratory syndrome [5,6]. Additionally, several studies also reported the utilization of the ROX index for predicting HFNC failure in COVID-19 patients who were admitted to the isolation areas and helped physicians decide when to intubate [7-9].

Therefore, this study aims to examine the accuracy of a ROX index in determining HFNC failure in COVID-19 patients associated with acute respiratory distress syndrome (ARDS) throughout the period of HFNC therapy. Additionally, we anticipate finding the best cut-off point for predicting HFNC failure when compared to the traditional cut-off point of the ROX index of 4.88.

**Methods**

This study will be conducted in a single-center manner as a retrospective observational cohort study.

**Study population**

The target population is adult patients with SAR-CoV2 infection who suffered from acute hypoxic respiratory failure (AHRF) and were treated with HFNC in the intensive care units (ICUs) and cohort wards of the Faculty of Medicine Vajira Hospital, Navamindradhiraj University, Bangkok, Thailand, between April 1 and August 30, 2021.

In order to be eligible to participate in this study, a subject must meet all of the following criteria:

- Adult patients aged equal to 18 years old or greater.

- A diagnosis of COVID-19 disease defined according to the positive result from the reverse transcription-polymerase chain reaction (RT-PCR) from a nasopharyngeal specimen and bilateral pulmonary infiltration on chest radiography.

- Patients commenced HFNC during the hospitalization.

A potential subject who meets any of the following criteria will be excluded from participation in this study:

- The patients with COVID-19 pneumonia who were initiated with an invasive mechanical ventilator before HFNC therapy or transferred to another hospital.

- Patient who signs for do-not-resuscitation orders.

**Sample size calculation**

The sample sizes are estimated parametrically based on the variance of the area under the receiver operating characteristic curve (AUC) and its marginal errors with a 95% confidence level for binormal assumption using the Hanley and McNeil formula [10]. The authors estimated sample sizes by using an AUC of 0.74 and a 95% confidence interval (CI) of 0.64-0.84 from the study by Roca O et al [5], a significance level of 0.05, and a power of the test at 80%. A minimum of 211 participants are required.

**Study parameters**

Data will be collected as the following:

- Independent variables: age, sex, height, weight, pre-existing comorbidities.

- At the initial phase of HFNC therapy, we will also collect the patient’s vital signs; including body temperature, heart rate (HR), mean arterial pressure (MAP), respiratory rate (RR), oxygen saturation (SpO2), fractional-inspired oxygen (FiO2), Flow of HFNC, severity score as measured by the Sequential Organ Failure Assessment (SOFA) score, and basic laboratory investigations, including complete blood count (CBC), serum creatinine, D-dimer, and C-reactive protein.

- We will collect RR, SpO2, and FiO2 of HFNC and calculate the ratio of the ROX index every 4 hours over the course of initiated-to-terminated HFNC therapy.

- The outcomes include the length of ICU stay, length of hospital stays, patients status on day 28 after HFNC treatment, and hospital mortality.

**Definition**

- COVID-19 patients will be defined according to a positive result by reverse transcription-polymerase chain reaction (RT-PCR) from nasopharyngeal specimens.

**-** High-flow nasal cannula failure (HFNC failure) will be defined as the subsequent needed for intubation and supported with invasive mechanical ventilation

- The ROX index score will be defined as the ratio of SpO2/FiO2(%) to respiratory rate (breaths/min) [5].

**Study procedures**

The data collection process will be started after the Institutional Review Board’s approval. Accessing, copying, and recording data from the medical record will be restricted to the research team. All data will be saved in an electronic file that can be accessed with a unique password.

**Data analysis**

We will present the descriptive statistics for continuous variables as mean and standard deviation (SD) or median and interquartile range (IQR) as appropriate. Categorical variables will be presented as frequencies and percentages. Comparing the results of the HFNC failure group and the HFNC success group, the Mann–Whitney U test will be used to compare continuous variables and Fisher's exact test for categorical variables.

Accuracy analyses of the ROX index for predicting the failure of HFNC therapy in COVID-19 patients associated with acute respiratory failure will be analyzed using receiver operating characteristic (ROC) and area under curve (AUC). The consideration cut-off points of the ROX index will be analyzed using Youden's index. A report with sensitivity, specificity, accuracy, positive predictive value (PPV), and negative predictive value (NPV) will be used. Statistical analyses will be performed using STATA version 16.0 (STATA Inc., College Station, TX, USA).

**Patients and public involvement**

This is an observational study in which all patients’ profiles, including medical records, are not publicly disclosed. The data will be extracted and analyzed anonymously.

**Ethics and dissemination**

The study will be conducted following the Declaration of Helsinki principles and Good Clinical Practice guidelines. Approval for this study will be obtained from the Vajira Institutional Review Board

**Discussion**

Currently, there are few studies demonstrating the use of the ROX index throughout HFNC therapy in predicting HFNC failure in COVID-19 patients. This study will help clarify whether or not the ROX index values calculated every 4 hours throughout the HFNC therapy can determine the HFNC failure group. Additionally, we will find the best cut-off point of the ROX index for predicting HFNC failure in COVID-19 patients.

**References**

1. Leasa D, Cameron P, Honarmand K, Mele T, Bosma KJ. Knowledge translation tools to guide care of non-intubated patients with acute respiratory illness during the COVID-19 Pandemic. Crit Care. 2021;25(1):22.

2. Alhazzani W, Møller MH, Arabi YM, Loeb M, Gong MN, Fan E, et al. Surviving Sepsis Campaign: guidelines on the management of critically ill adults with Coronavirus Disease 2019 (COVID-19). Intensive Care Med. 2020;46(5):854-87.

3. Tobin MJ. Basing Respiratory Management of COVID-19 on Physiological Principles. Am J Respir Crit Care Med. 2020;201(11):1319-20.

4. Pandya A, Kaur NA, Sacher D, O'Corragain O, Salerno D, Desai P, et al. Ventilatory Mechanics in Early vs Late Intubation in a Cohort of Coronavirus Disease 2019 Patients With ARDS: A Single Center's Experience. Chest. 2021;159(2):653-6.

5. Roca O, Messika J, Caralt B, García-de-Acilu M, Sztrymf B, Ricard JD, et al. Predicting success of high-flow nasal cannula in pneumonia patients with hypoxemic respiratory failure: The utility of the ROX index. J Crit Care. 2016;35:200-5.

6. Roca O, Caralt B, Messika J, Samper M, Sztrymf B, Hernández G, et al. An Index Combining Respiratory Rate and Oxygenation to Predict Outcome of Nasal High-Flow Therapy. Am J Respir Crit Care Med. 2019;199(11):1368-76.

7. Suliman LA, Abdelgawad TT, Farrag NS, Abdelwahab HW. Validity of ROX index in prediction of risk of intubation in patients with COVID-19 pneumonia. Adv Respir Med. 2021;89(1):1-7.

8. Goury A, Moussanang JA, Bard M, Champenois V, Julien G, Dupont V, et al. Predictive factors associated with high-flow nasal cannula success for COVID-19-related acute hypoxemic respiratory failure. Health Sci Rep. 2021;4(2):e287.

9. Goh KJ, Chai HZ, Ong TH, Sewa DW, Phua GC, Tan QL. Early prediction of high flow nasal cannula therapy outcomes using a modified ROX index incorporating heart rate. J Intensive Care. 2020;8:41.

10. Hajian-Tilaki K. Sample size estimation in diagnostic test studies of biomedical informatics. J Biomed Inform. 2014;48:193-204.
